# Podocalyxin regulates astrocytoma cell invasion and survival against temozolomide

**DOI:** 10.3892/etm.2013.957

**Published:** 2013-02-15

**Authors:** HAO WU, LIANG YANG, DAGUANG LIAO, YUDAN CHEN, WEI WANG, JIASHENG FANG

**Affiliations:** 1 Department of Neurosurgery, Xiangya Hospital, Central South University, Changsha, Hunan 410008;; 2 Departments of Neurosurgery, The Third Xiangya Hospital, Central South University, Changsha, Hunan 410013;; 3 Emergency, The Third Xiangya Hospital, Central South University, Changsha, Hunan 410013;; 4 Department of Neurology, The Second Xiangya Hospital, Central South University, Changsha, Hunan 410011, P.R. China

**Keywords:** astrocytoma, glioblastoma, podocalyxin, invasion, cell survival, temozolomide, chemoresistance, phosphatidy linositol 3-kinase/Akt pathway

## Abstract

Increased podocalyxin (PODXL) expression has been associated with a subset of aggressive types of cancer. To the best of our knowledge, the effect of PODXL on astrocytoma cell invasion and survival against chemotherapy agent was investigated for the first time in the present study. Overexpression and knockdown of PODXL were respectively performed in SW1783 (grade III astrocytoma) and U-87 (grade IV astrocytoma; gliobalstoma) cells. PODXL overexpression in SW1783 cells significantly increased cell invasion, matrix metalloproteinase-9 (MMP-9) expression, cell survival against temozolomide-induced apoptotic stress, and phosphorylation of Akt at serine 473 (ser473), which was abolished by the selective phosphatidylinositol 3-kinase (PI3K) inhibitor LY294002 (LY). Knockdown of PODXL in U-87 cells significantly decreased cell invasion, MMP-9 expression, cell survival against temozolomide, and phosphorylation of Akt at serine 473 (ser473), which was further decreased by LY treatment. In conclusion, in the present study it was demonstrated that PODXL promotes astrocytoma cell invasion, potentially through the upregulation of MMP-9 expression in a PI3K-dependent manner. Additionally, PODXL was shown to promote astrocytoma cell survival against temozolomide-induced apoptotic stress by enhancing the activation of the PI3K/Akt survival signaling pathway. This study provides novel insights into the molecular mechanisms underlying astrocytoma progression, cell survival and chemoresistance, and suggests that PODXL may be a potential target for overcoming chemoresistance in astrocytomas.

## Introduction

Astrocytic tumors are the most common tumors of the central nervous system (CNS) and are categorized into diffuse astrocytomas [World Health Organization (WHO) grade II], anaplastic astrocytomas (WHO grade III) and glioblastomas (WHO grade IV) (1). More than 51,000 individuals are diagnosed with a primary brain tumor in the United States each year, and for those with astrocytoma, ∼75% succumb to the disease within 5 years of diagnosis (2). Although surgery, radiation and chemotherapy have improved the length of survival, astrocytoma mortality remains high. Particularly, the overall survival rate of glioblastoma patients was only 17.7% at one year, and 3.3% at two years (3,4). Therefore, novel strategies to treat astrocytoma, particularly glioblastoma, are urgently required. However, the mechanisms of malignant progression of astrocytic tumors have not been completely resolved.

Podocalyxin (PODXL) is a highly glycosylated and sialylated transmembrane protein, and a CD34 ortholog normally expressed on hematopoietic stem cells, hemangioblasts, vascular endothelial cells, podocytes and a subset of neural progenitors (5). Recently, increased PODXL expression has been associated with a subset of aggressive cancers including acute myeloid and lymphoid leukemia, myeloid sarcomas, as well as certain breast, liver, pancreatic and kidney tumors (5,6). The clinical significance of PODXL in cancer progression has been investigated in numerous tumor types, including breast, colon and uterine carcinoma. In uterine endometrioid adenocarcinoma, PODXL expression is correlated with tumor grade (7), while its overexpression is an independent indicator of poor outcome in breast and colorectal carcinoma (8,9). PODXL also reportedly leads to increased *in vitro* migration and invasion, increased matrix metalloproteinase (MMP) expression, and increased activation of phosphatidylinositol 3-kinase (PI3K) in breast and prostate cancer cells (10). Thus, PODXL may play a critical role in cancer development and aggressiveness. A recent study reported that PODXL expression was detected on the surface of 42.9% of anaplastic astrocytoma samples and 54.8% of glioblastoma samples, suggesting that PODXL may be associated with the malignant progression of astrocytic tumors (11). However, the role of PODXL in astrocytoma progression remains to be fully elucidated. In the present study, the effect of PODXL on astrocytoma cell invasion and survival against a chemotherapy agent was investigated.

## Materials and methods

### Cells lines, plasmids and reagents

The human astrocytoma cell lines SW1783 and U-87 were purchased from the American Tissue Culture Collection (ATCC, Rockville, MD, USA). Human full length PODXL cDNA was subcloned into pcDNA 3.1 expression vector. Human PODXL shRNA plasmid (RHS3979-98487921) and pLKO.1 empty plasmid (RHS4080) were purchased from Open Biosystems, Inc. (Huntsville, AL, USA). Anti-PODXL (3D3; 39-3800) antibody was purchased from Life Technologies (Carlsbad, CA, USA). Anti-MMP-9 (sc-13520), anti-Akt (ser473; sc-24500) and anti-P-Akt (ser473; sc-101629) antibodies were purchased from Santa Cruz Biotechnology, Inc. (Santa Cruz, CA, USA). All the secondary antibodies were purchased from Jackson ImmunoResearch Laboratories, Inc. (West Grove, PA, USA). The DeadEnd™ Fluorometric TUNEL system was purchased from Promega (Madison, WI, USA). SuperFect^®^ transfection reagent was purchased from Qiagen (Valencia, CA, USA). Temozolomide, LY294002 (LY) and all the chemicals of reagent grade were purchased from Sigma (St. Louis, MO, USA).

### Transfection and lentiviral transduction

The PODXL expression construct was transfected into SW1783 and U-87 cells using SuperFect^®^ transfection reagent according to the manufacturer’s instructions. Pools of stable transductants were generated via selection with G418 (800 *μ*g/ml) using the manufacturer’s protocol. Lentiviral transduction was performed in the SW1783 and U-87 cells. Pools of stable transductants were generated via selection with puromycin (5 *μ*g/ml).

### In vitro cell invasion assay

Transwell^®^ cell invasion assays (Corning Life Sciences, Lowell, MA, USA) were performed. Briefly, Transwell^®^ cell culture chambers with 8-*μ*m pore size (BD Biosciences, Bedford, MA, USA) for 24-well plates were coated with 50 *μ*l Matrigel (BD Biosciences; 10 mg/ml; diluted 1:3 in RPMI-1640). The SW1783 and U-87 cells were seeded in the upper chamber at 5×10^5^ cells/well in RPMI-1640 serum-free medium. Complete medium (600 ml) was added to the lower chamber. The cells were treated with LY (50 *μ*M) and allowed to migrate for 24 h followed by fixation and staining with crystal violet. The invasive cells were counted in 10 random fields/chamber under a microscope. Each experiment was repeated three times in triplicate.

### Western blot analysis

Immunoblotting was performed with the respective antibodies. Briefly, cells were dissolved in 250 *μ*l 2X SDS loading buffer (62.5 mM Tris-HCl, pH 6.8, 2% SDS, 25% glycerol, 0.01% bromophenol blue and 5% 2-mercaptoethanol), and incubated at 95°C for 10 min. Equal amount of proteins for each sample were separated by 10% SDS-polyacrylamide gel and blotted onto a polyvinylidene difluoride microporous membrane (Millipore, Billerica, MA, USA). Membranes were incubated for 1 h with a 1/1000 dilution of the primary antibody (1/10000 for 3D3 PODXL blotting), and then washed and revealed using secondary antibodies with horseradish peroxidase conjugate (1/5000, 1 h). Peroxidase was revealed using an ECL kit (GE Healthcare, Piscataway, NJ, USA). Proteins were quantified before being loaded onto the gel, and equal loading of extracts was verified by Ponceau coloration.

### Measurement of apoptosis by TUNEL (terminal deoxynucleotidyl-transferase-mediated nick-end labeling) assay

The TUNEL assay was performed using the DeadEnd™ Fluorometric TUNEL system following the instructions provided by Promega. Cells were treated with temozolamide (100 *μ*M) in the presence or absence of LY (50 *μ*M) for up to 8 h. Apoptotic cells exhibited a strong nuclear green fluorescence that was detected using a standard fluorescein filter. All the cells stained with DAPI exhibited a strong blue nuclear fluorescence. The slides were observed under a fluorescent microscope with relative apoptotic cells determined by counting the TUNEL-positive cells in five random fields (magnification, ×100) for each sample.

### Statistical analysis

Statistical analyses were performed with SPSS for Windows 10.0. Data values were expressed as the mean ± standard deviation (SD). Comparisons of means among multiple groups were performed with one-way ANOVA followed by post hoc pairwise comparisons using the least significant difference method. The significance level of this study was set at a two-tailed P=0.05.

## Results

### Effect of PODXL overexpression and knockdown on astrocytoma cell invasion and MMP-9 expression

**Figure 1:**
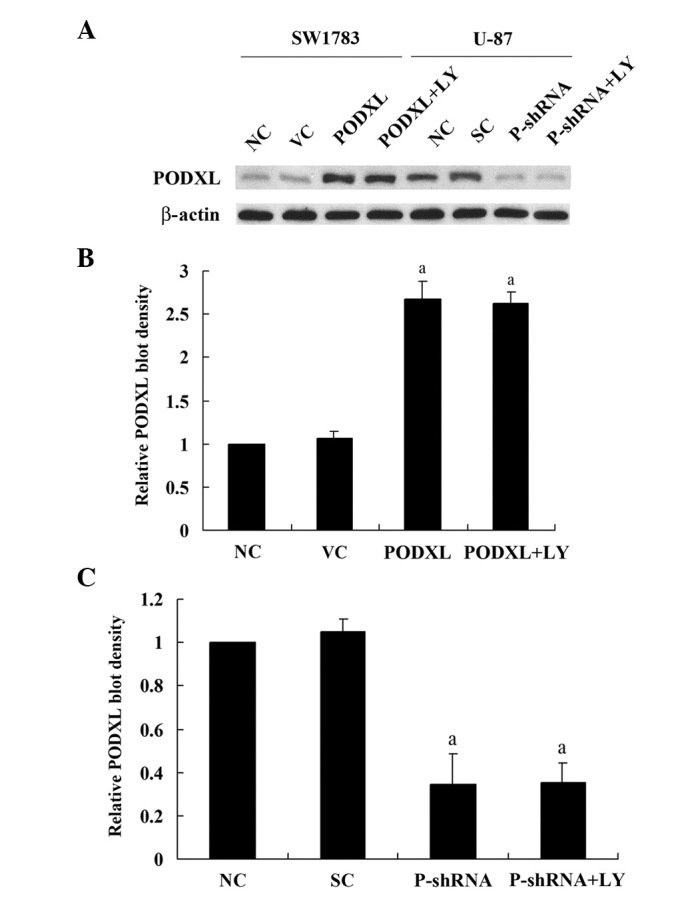
Western blot analysis of podocalyxin (PODXL) expression in SW1783 and U-87 astrocytoma cells. (A) In SW1783 cells, PODXL expression was analyzed in normal control cells (NC), cells stably transfected with empty pcDNA3 vector (VC) and cells stably transfected with pcDNA3-PODXL expression vector (PODXL) with or without LY294002 (LY; 50 *μ*M) treatment by western blotting. In U-87 cells, PODXL expression was analyzed in NC, cells stably transduced with scramble control shRNA (SC) and cells stably transduced with PODXL-shRNA (P-shRNA) with or without LY (50 *μ*M) treatment by western blotting. β-actin blotting was used as a loading control. Density of PODXL was normalized against that of β-actin to obtain a relative blot density, which was expressed as fold change to the relative blot density of NC (designated as 1) in (B) SW1783 and (C) U-87 cells. ^a^P<0.05 compared with (B) NC and VC or (C) SC.

As shown in Fig. 1, SW1783 (grade III astrocytoma) cells had a relatively low constitutive expression of PODXL compared with U-87 (grade IV astrocytoma; gliobalstoma) cells. Thus, to investigate the functional role of PODXL in astrocytoma cells, we stably transfected SW1783 cells with PODXL expression vector to overexpress PODXL, and stably transduced U-87 cells with PODXL-shRNA to knock down PODXL. Compared with the controls, PODXL was overexpressed by >2.5-fold in SW1783 cells, and the endogenous PODXL level was knocked down ∼70% in U-87 cells. Selective PI3K inhibitor LY showed no effect on PODXL expression in either cell line.

**Figure 2:**
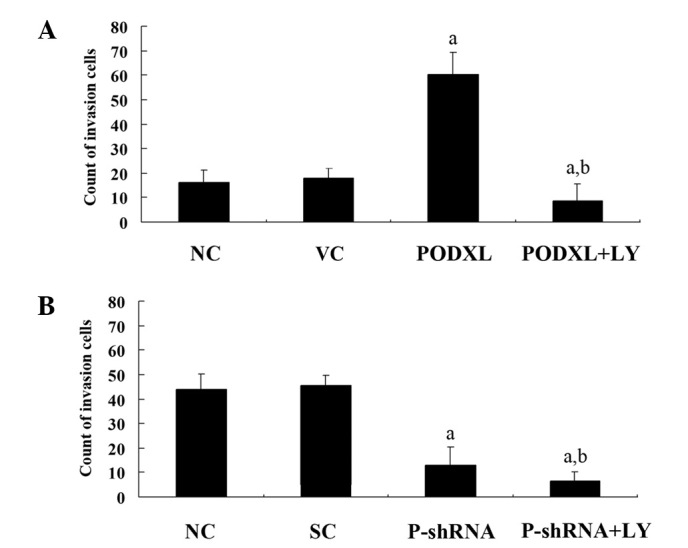
*In vitro* cell invasion by SW1783 and U-87 cells. A) In SW1783 cells, *in vitro* cell invasion assays were performed in normal control cells (NC), cells stably transfected with empty pcDNA3 vector (VC) and cells stably transfected with pcDNA3-podocalyxin expression vector (PODXL) with or without LY294002 (LY; 50 *μ*M) treatment. (B) In U-87 cells, *in vitro* cell invasion assays were performed in NC, cells stably transduced with scramble control shRNA (SC) and cells stably transduced with podocalyxin-shRNA (P-shRNA) with or without LY (50 *μ*M) treatment. Invasion cell numbers were counted and the cell invasion level was shown as fold change of invasion cell number to that of NC (designated as 1). ^a^P<0.05 compared with (A) NC and VC or (B) SC; ^b^P<0.05 compared with (A) PODXL or (B) P-shRNA.

**Figure 3:**
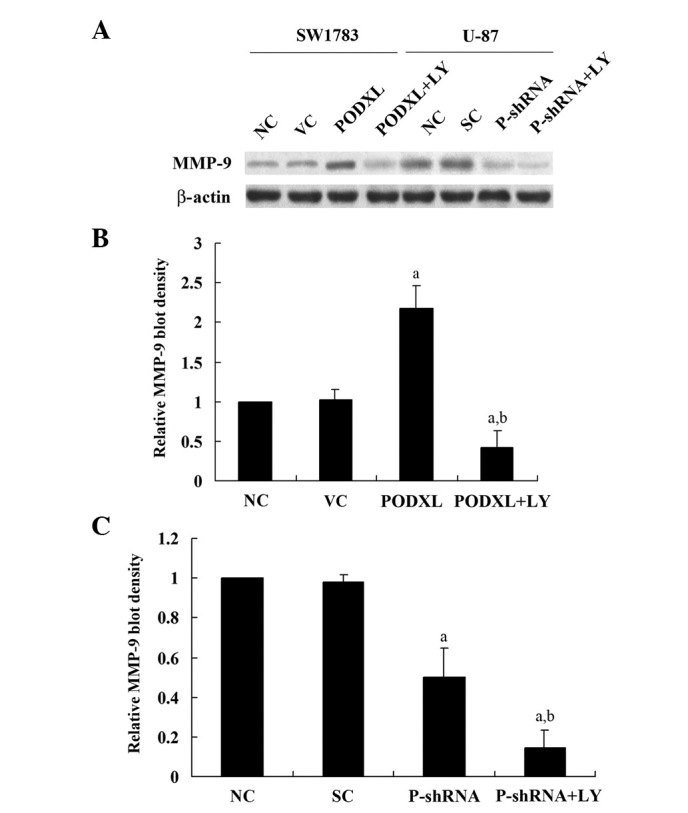
Western blot analysis of matrix metalloproteinase-9 (MMP-9) expression in SW1783 and U-87 cells. (A) In SW1783 cells, MMP-9 expression was analyzed in normal control cells (NC), cells stably transfected with empty pcDNA3 vector (VC) and cells stably transfected with pcDNA3-podocalyxin expression vector (PODXL) with or without LY294002 (LY; 50 *μ*M) treatment was analyzed by western blotting. (B) In U-87 cells, MMP-9 expression was analyzed in NC, cells stably transduced with scramble control shRNA (SC) and cells stably transduced with podocalyxin-shRNA (P-shRNA) with or without LY (50 *μ*M) treatment by western blotting. β-actin blotting was used as a loading control. Density of MMP-9 was normalized against that of β-actin to obtain a relative blot density, which was expressed as fold change to the relative blot density of NC (designated as 1) in (B) SW1783 and (C) U-87 cells. ^a^P<0.05 compared with (B) NC and VC or (C) SC; ^b^P<0.05 compared with (B) PODXL or (C) P-shRNA.

PODXL has been reported to promote tumor cell invasion through MMPs (10). To investigate the effect of PODXL on astrocytoma cell invasion, we performed *in vitro* cell invasion assays and examined the MMP-9 expression level in the two cell lines. As shown in Fig. 2, PODXL overexpression in SW1783 cells increased cell invasion by ∼4-fold compared with that of the controls, and this increase was eradicated by LY. By contrast, PODXL knockdown in U-87 cells decreased cell invasion by ∼3-fold compared with the controls, and this was further decreased by LY treatment. Similar trends were observed with MMP-9 expression (Fig. 3). These results suggest that PODXL promotes astrocytoma cell invasion, potentially by upregulating MMP-9 expression in a PI3K-dependent manner.

### Effect of PODXL overexpression and knockdown on astrocytoma cell survival against temozolamide-induced apoptosis

**Figure 4:**
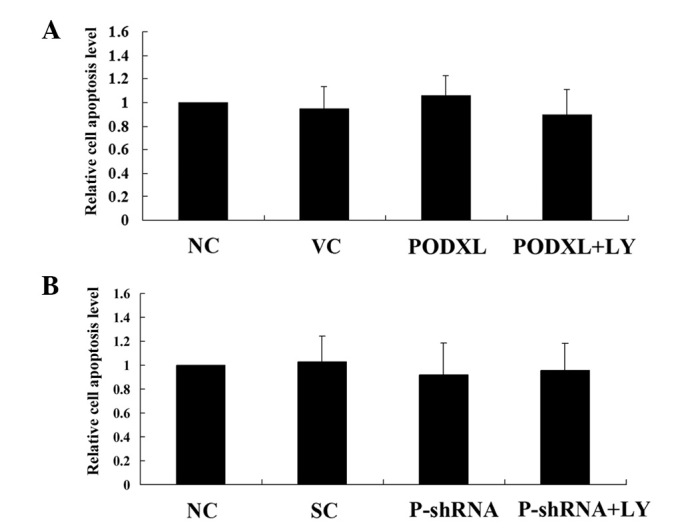
Cell apoptosis in SW1783 and U-87 cells under normal culture conditions. (A) SW1783 and (B) U-87 cells were cultured under normal culture conditions for 8 h. The cell apoptosis rate was determined as as the percentage of TUNEL-positive cells in total cells at the 8th h. In SW1783 cells, TUNEL assays were performed in normal control cells (NC), cells stably transfected with empty pcDNA3 vector (VC) and cells stably transfected with pcDNA3-podocalyxin expression vector (PODXL) with or without LY294002 (LY; 50 *μ*M) treatment. In U-87 cells, TUNEL assays were performed in NC, cells stably transduced with scramble control shRNA (SC) and cells stably transduced with podocalyxin-shRNA (P-shRNA) with or without LY (50 *μ*M) treatment. The cell apoptosis level was shown as fold change to that of NC (designated as 1).

**Figure 5:**
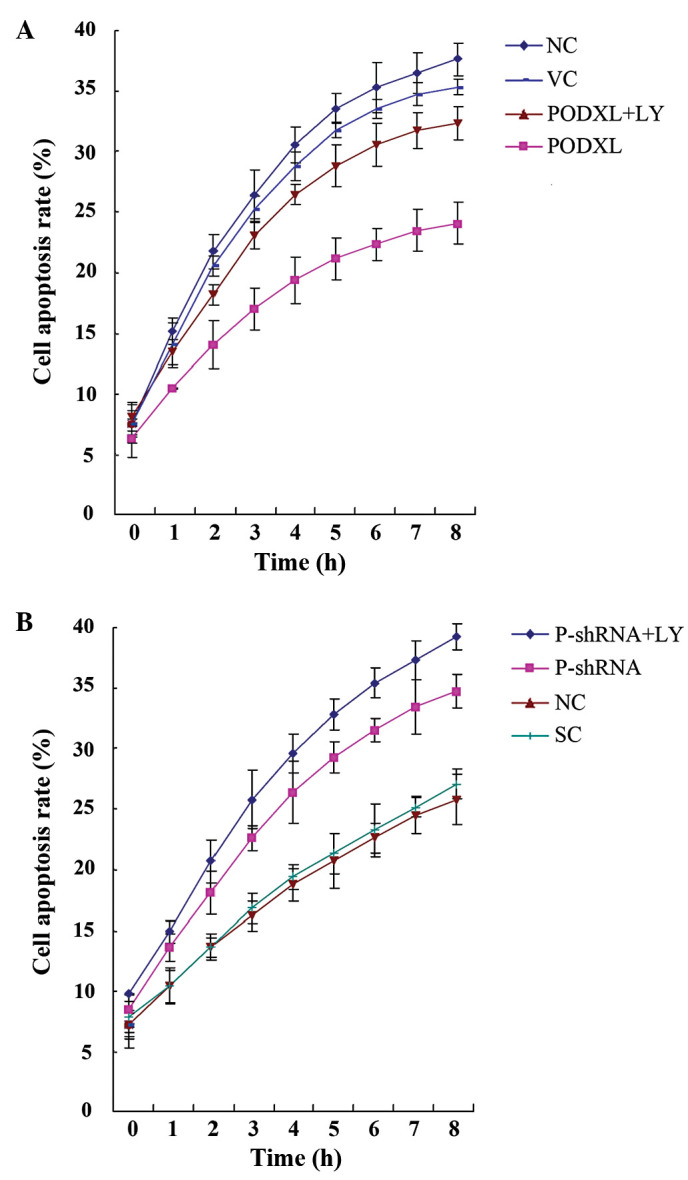
Cell apoptosis in SW1783 and U-87 cells treated with temozolomide. (A) SW1783 and (B) U-87 cells were treated with 100 *μ*M of temozolomide for 8 h. In SW1783 cells, TUNEL assays were performed in normal control cells (NC), cells stably transfected with empty pcDNA3 vector (VC) and cells stably transfected with pcDNA3-podocalyxin expression vector (PODXL) with or without LY294002 (LY; 50 *μ*M) treatment. In U-87 cells, TUNEL assays were performed in NC, cells stably transduced with scramble control shRNA (SC) and cells stably transduced with podocalyxin-shRNA (P-shRNA) with or without LY (50 *μ*M) treatment. The cell apoptosis rate was shown as the percentage of TUNEL-positive cells in total cells.

PODXL reportedly promotes the metastatic potential of tumor cells. Since tumor cell survival is critical for metastasis (12), we next examined the effect of PODXL on astrocytoma cell survival against apoptotic stress. As shown in Fig. 4, PODXL overexpression and knockdown with or without LY treatment did not significantly alter the apoptosis rate of astrocytoma cells in normal culture conditions. Then, the cells were treated with 100 *μ*M of temozolomide, an apoptosis-inducing chemotherapeutic agent used to treat high-grade astrocytoma. In SW1783 cells treated with temozolamide, PODXL overexpression significantly reduced cell apoptosis compared with that in the controls, and this reduction was reversed by LY (Fig. 5). In U-87 cells, PODXL knockdown significantly increased cell apoptosis in the presence of temozolamide. LY treatment further increased the apoptosis in the PODXL-knocked down cells (Fig. 5).These results suggest that PODXL promotes astrocytoma cell survival against temozolamide in a PI3K-dependent manner.

### Effect of PODXL overexpression and knockdown on the PI3K/Akt survival signaling pathway in astrocytoma cells

**Figure 6:**
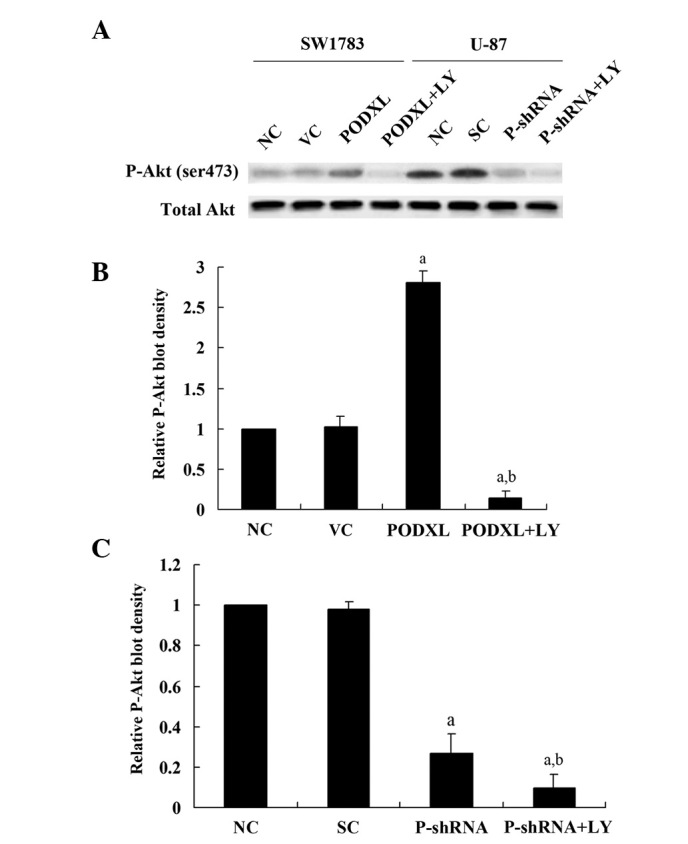
Western blot analysis of phosphorylated Akt (P-Akt) level in SW1783 and U-87 cells. (A) In SW1783 cells, the level of total Akt and P-Akt at serine 473 (ser473) was analyzed in normal control cells (NC), cells stably transfected with empty pcDNA3 vector (VC) and cells stably transfected with pcDNA3-podocalyxin expression vector (PODXL) with or without LY294002 (LY; 50 *μ*M) treatment by western blotting. (B) In U-87 cells, the level of total Akt and P-Akt at serine 473 (ser473) was analyzed in NC, cells stably transduced with scramble control shRNA (SC) and cells stably transduced with podocalyxin-shRNA (P-shRNA) with or without LY (50 *μ*M) treatment by western blot analysis. P-Akt (ser473) and total Akt blots were measured by densitometry. Density of the P-Akt (ser473) blot was normalized against that of total Akt to obtain a relative P-Akt (ser473) blot density, which was expressed as fold change to the relative P-Akt (ser473) blot density of NC (designated as 1). ^a^P<0.05 compared with (B) NC and VC or (C) SC; ^b^P<0.05 compared with (B) PODXL or (C) P-shRNA.

Since PODXL showed a protective effect on astrocytoma cells against temozolomide-induced apoptotic stress in a PI3K-dependent manner (Fig. 5), we investigated the effect of PODXL on the PI3K/Akt survival signaling pathway in astrocytoma cells. In SW1783 cells, PODXL overexpression significantly increased phosphorylation at serine 473 (ser473) of Akt, which is required for full activation of Akt (Fig. 6). LY treatment totally eradicated the effect of PODXL over-expression. In U-87 cells, PODXL knockdown decreased phosphorylation at serine 473 (ser473) of Akt by >3-fold compared with the control level (Fig. 6), which was further decreased by LY treatment. Taken together, these findings suggest that PODXL enhances the activation of the PI3K/Akt signaling pathway and, thereby, promotes astrocytoma cell survival against apoptotic stress.

## Discussion

PODXL reportedly increases the aggressive phenotype of numerous types of cancer, including acute myeloid and lymphoid leukemia, myeloid sarcomas, as well as certain breast, liver, pancreatic and kidney tumors (13,14). To the best of our knowledge, the effect of PODXL on astrocytoma cell invasion and survival against chemotherapy agent was investigated for the first time in the present study

We examined several astrocytoma cell lines and found that while PODXL was amply expressed in U-87 cells, it was expressed at a low level in SW1783 cells. Thus, overexpression and knockdown of PODXL were respectively performed in the two cell lines.

Sizemore *et al* (10) reported that PODXL overexpression increased the *in vitro* invasive potential of breast and prostate cancer cells and led to increased MMP-9 expression and enhanced PI3K activity in the cells. Similar results in astrocytoma cells were found in the present study. Additionally, our findings that the selective PI3K inhibitor LY eradicated the effect of PODXL overexpression and extended the effect of PODXL knockdown, suggest that PODXL promotes invasion and MMP-9 expression in astrocytoma cells by a PI3K-dependent mechanism.

Besides invasion potential, cell viability against apoptotic stress is an additional important characteristic of metastatic tumor cells (12). To the best of our knowledge, the effect of PODXL on astrocytoma cell viability/survival against chemotherapeutic agent-induced apoptotic stress was investigated for the first time in the present study. Temozolomide alkylates/methylates DNA, which damages DNA and triggers the death of tumor cells (15). Borges *et al* (16) showed that the IC_50_ of temozolomide on glioblastoma cells was >300 *μ*M. Thus, in the present study, we used a relatively small concentration of temozolomide (100 *μ*M) to induce apoptotic stress without killing most of the cells. Our results showed that PODXL knockdown significantly increased cell apoptosis in the presence of temozolamide, suggesting that PODXL may be a potential target for overcoming chemoresistance in astrocytomas, particularly, glioblastomas. However, it still remains unclear whether PODXL knockdown would impact astrocytoma cell survival against other types of chemotherapy agents. As a result, further studies with more types of chemotherapy agents and astrocytoma cell lines are required.

In conclusion, we demonstrated that PODXL promotes astrocytoma cell invasion, potentially through upregulating MMP-9 expression in a PI3K-dependent manner. Additionally, PODXL was shown to promote astrocytoma cell survival against temozolomide-induced apoptotic stress by enhancing the activation of the PI3K/Akt survival signaling pathway. This study provides novel insights into the molecular mechanisms underlying astrocytoma progression, cell survival and chemoresistance.
